# Design of a multipurpose sample cell holder for the Diamond Light Source high-throughput SAXS beamline B21

**DOI:** 10.1107/S1600577520013831

**Published:** 2021-01-01

**Authors:** Charlotte Jennifer Chante Edwards-Gayle, Nikul Khunti, Ian W. Hamley, Katsuaki Inoue, Nathan Cowieson, Robert Rambo

**Affiliations:** a B21, Diamond Light Source Limited, Chilton, Didcot, Oxfordshire OX11 0DE, United Kingdom; b Department of Chemistry, Food and Pharmacy, University of Reading, Whiteknights, Reading, Berkshire RG6 6AD, United Kingdom

**Keywords:** BioSAXS, high-throughput SAXS, multipurpose sample holders

## Abstract

The design of a multipurpose sample cell holder for the high-throughput (HT) beamline B21 is presented. The device is compatible with the robot bioSAXS sample changer currently installed on BM29, ESRF, and P12 Petra IV synchrotrons. This work presents an approach that uses 3D-printing to make hardware alterations which can expand the versatility of HT beamlines at low cost.

## Abstract 2

Beamline B21 at Diamond Light Source is a high-throughput beamline that focuses primarily on the fast measurement of solutions. Here 3D-printing is used to expand the versatility of sample states that can be measured on B21.

## 1. Introduction   

Small-angle X-ray scattering (SAXS) measures the nanoscale structure of many materials in all physical states of matter, with applications to biological systems including proteins, peptides and DNA, as well as other soft materials such as polymers and colloids. SAXS can be high-throughput (HT) and highly automated at synchrotron beamlines, typically accommodating a single or selective subset of sample environments. The restriction in sample environments standardizes the sample handling at the beamline, thereby increasing the speed and number of samples that can be processed and measured, but consequently limits the beamline to a specific subset of sample types. In order to accommodate different sample types (*e.g.* gels or waxes), alterations to the hardware of the beamline must be made. Ideally, if any alterations are made, the reconfiguration of the beamline must have a low down-time, be cost-effective and not affect the standard operating environment of the beamline.

To minimize beamline down-time, one option is to create sample holders compatible with the standard beamline configuration. Creating sample holders can be a limiting factor; however, the proliferation of 3D-printing has led to a variety of 3D-CAD software, enabling an inexpensive and low-effort method to adapt sample environments to HT beamlines. Thus, increasing the adaptability of HT beamlines expands the instrumentation capabilities and likewise increases access of HT measurements to a larger scientific community.

Here, we focus on the development and design of a multipurpose sample (MPS) cell, compatible with the HT SAXS beamline, B21, Diamond Light Source (Cowieson *et al.*, 2020 ▸). The MPS cell is designed using readily available 3D-printing software and is made *in situ* using a stereolithography (SLA) 3D printer. The window material is optimized to produce minimal background scattering as well as ease of use. The cell holder is compatible with weak and strong hydro­gels and also viscous liquids, precipitates, powders and solids.

Finally, we present representative SAXS data collected using this sample holder and display the different sample types that can now be measured at B21. This sample cell is compatible with other HT SAXS beamlines including P12 (Petra IV, Germany; Blanchet *et al.*, 2015 ▸) and BM29 (ESRF, France; Pernot *et al.*, 2013 ▸). These methods for design can be applied to other sample systems and specificities, enabling users to be able to conveniently measure a greater variety of sample types whilst not incurring large costs.

## 2. Development of the MPS cell   

### 2.1. Design of the MPS cell   

The B21 sample environment is provided by the Arinax sample handling robot (SHR) (Round *et al.*, 2015 ▸). The SHR contains the sample exposure unit (SEU) which maintains the sample capillary in a vacuum environment. At B21, data collection is achieved through an in-vacuum detector with only a single silicon-nitride window separating the beamline from the main synchrotron storage ring. Therefore, instrumentation background scatter is minimal and provided predominantly by the sample cell window material. The capillary system is designed to support liquid samples. Therefore, to provide additional capabilities for measuring non-liquid samples, a new sample cell (MPS) was designed to be compatible with the SEU that takes advantage of the low-background in-vacuum configuration. The MPS cell was developed using *Autodesk Fusion 360* and *OpenScad* 3D-printing software. Technical drawings (Figs. S1 and S2 of the supporting information) provide more detail about the different size and orientation parameters used to make these devices compatible with the SEU at B21 (Round *et al.*, 2015 ▸). The cell consists of two principal components: an outer sample pod compatible with the SEU [Figs. S4(*a*) and 2] and the sample stick [Fig. S4(*c*)].

### 2.2. Window frame aperture   

The sample stick has a tapered, asymmetric shape that is complementary to the insertion pathway of the sample pod [Fig. S4(*a*)]. The triangular tapered tip and square base of the sample stick [Fig. S4(*c*)] were designed to aid the alignment and reproducibility in positioning of the sample as the stick is placed inside the sample cell. The sample pod is capped internally by a threaded ‘alignment plug’ (Fig. S6) that receives the triangular tapered tip of the sample stick.

The sample stick has a length of 61 mm and a width of 7.8 mm (Figs. S4 and S5); the tip is triangular to insert into the top of the sample pod and prevent twisting. A 10.2 mm × 2 mm hole in the insert is where the sample is placed. The maximum sample path length is 1.7 mm.

A Formlabs Form 2 SLA 3D printer was used to print prototypes (Fig. S5) in methyl acrylic acid esters and photoinitator. Prototypes were used to identity and refine the design. Some issues identified through the design phase were (1) how to maintain reproducibility in sample position as the sample stick is used multiple times, (2) design of the opening on the sample pod which will support the vacuum windows and (3) ease of applying highly viscous, glassy samples to the sample space on the sample stick. Prototype cells were capable of holding the vacuum when placed within the SEU using polyimide (Kapton) windows attached to a simple printed frame. After the final design, the sample pod was manufactured in stainless steel to increase durability and allow for heat conduction for thermal stability by the SEU.

**Figure 1:**
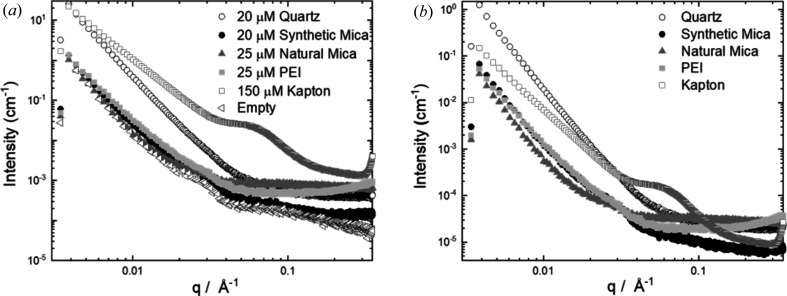
(*a*) Background of potential window materials and (*b*) those materials normalized to thickness. Synthetic mica windows were selected for the sample pod owing to low background scatter.

Further optimization of the sample pod was focused on the choice of window material. Polyimide (Kapton) and mica are robust materials routinely used as X-ray windows in SAXS. However, polyimide has a notable scattering feature: a near-scattering vector of 0.4 Å^−1^, and we therefore considered alternative materials to use as the sample pod window (Lurio *et al.*, 2007 ▸; Wang *et al.*, 2012 ▸). Fig. 1 ▸ shows the X-ray scattering profiles of the various materials tested normalized to sample thickness. Window materials were mounted onto a specialized cell with a single frame of window material. This cell was used due to the ease of mounting the window material compared with the MPS sample pod. Synthetic mica windows were selected due to low-background scatter (Masunaga *et al.*, 2013 ▸).

B21 is a bending-magnet beamline with a beam size (full width at half-maximum) of 1102 µm × 240 µm at the sample position. The initial window design matched the size of the sample aperture on the sample stick. The horizontal divergence properties of the beam led to considerable parasitic scattering from the resin used to make the sample stick. To minimize the parasitic scattering, it was necessary to make a window with a small aperture of ∼2 mm using a high-electron-density material such as stainless steel. This eliminated the stray, parasitic scattering from the sample stick resin reducing the overall background scatter from the sample cell. The final window design consists of a stainless-steel frame, with a 25 µm-thick synthetic mica sheet adhered to the frame with double-sided tape. The frame was sealed in place with ep­oxy. This combination of synthetic mica and stainless-steel frame provides the lowest possible background for the sample environment (Fig. 1 ▸).

## 3. Data collection of different sample types   

### 3.1. MPS cell data collection   

**Figure 2:**
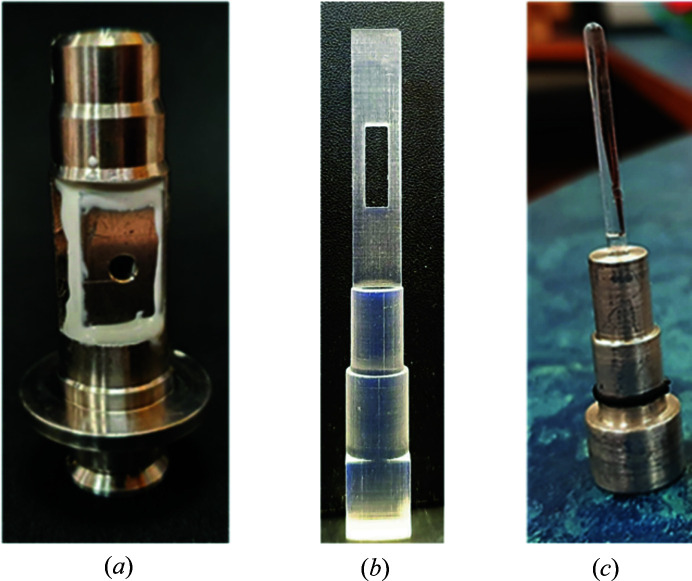
Finished designs of (*a*) machined sample pod (with the steel plate and mica windows glued) with (*b*) 3D-printed sample stick and (*c*) capillary insert.

The cell is compatible with a number of different sample types including powders, films, solids, viscous liquids (which cannot uptake by standard robot injection) and weak to strong hydro­gel/gel-like substances. For solid substances and films, it is possible to mount or wedge them into the cell with no further support. However, for weak gels and viscous liquids a different approach is required. Here the samples must be enclosed. To overcome this, when samples are in this state, we can enclose them using Kapton tape. Kapton tape was selected due to ease of use, durability and to make sample sticks more recyclable (Fig. 2 ▸). The time taken to change the setup between liquid handling mode and MPS mode and vice versa takes 10 min. Currently, samples are manually loaded in MPS mode, taking around 5 min to run each sample. Samples are maintained at atmospheric pressure during data collection.

To test the quality and show the versatility of the sample holder, different example substances (Table 1 ▸) were measured using the MPS cell under normal setup conditions.

**Figure 3:**
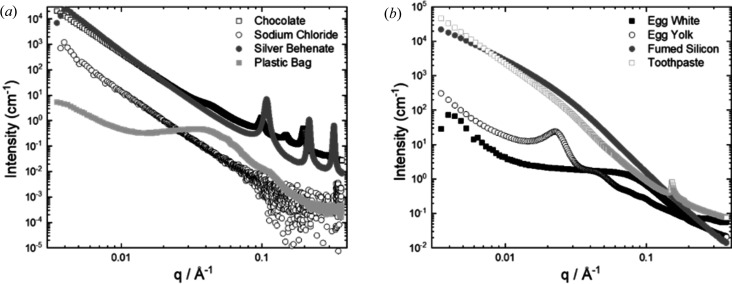
SAXS data collected from the MPS cell for different sample types, ranging from thick liquids to solid films and powders. The peaks correspond to ordering in the samples.

The data (Fig. 3 ▸) from an array of different sample types have a low signal-to-noise ratio and can detect important structural information from the data.

Overall, the MPS cell expands the range of different sample types that can be measured at B21, Diamond Light Source, increasing the versatility of the beamline. Users now have the option of measuring solutions, solids, viscous liquids, powders, pastes, films and hydro­gel/gel-like substances. The methods of design presented here (3D-printing of prototypes) can be used to design other cells as means for low cost hardware modification. Future work will involve automating the system in-line with the HT ethos of B21.

## 4. Materials and methods   

Polyethyl­ene imide was supplied from goodfellow, synthetic mica sheets by Great Wall Mineral (China), silicon nitride membrane mounts by Silson LtD and quartz windows by ATOCK Inc.

The MPS cell prototypes were designed using the 3D-modelling software *OpenScad* (https://www.openscad.org/) and *Autodesk Fusion 360* (https://www.autodesk.com/products/fusion-360).

Designs generated from *OpenScad* and *Fusion360* were 3D-printed using the *Formlabs Preform* printing software, on a Formlabs Form 2 printer in either Resin V2 or Resin V4.

Small-angle X-ray scattering was carried out on B21, Diamond Light Source. Samples were either mounted onto the MPS sample stick or enclosed in Kapton tape in the sample hole and then placed inside the MPS sample pod. The sample-to-detector distance was 3.712 m, operating at 13.018 keV. SAXS was performed at B21 operating at a 3.7 m camera length, 13.02 keV. Samples were manually loaded and exposed for 1 s, collecting 28 frames at 20°C. Data were collected using a Dectris Eiger 4M detector. The background was manually subtracted using *ScÅtter* (https://www.bioisis.net/, R. P. Rambo).

## Supplementary Material

Supporting figures. DOI: 10.1107/S1600577520013831/ju5015sup1.pdf

## Figures and Tables

**Table 1:** Methods for loading different example substances into the MPS cell

State	Example material	Method for loading into MPS cell
Solid	Chocolate	Wedged in, no Kapton windows
Solid film	Plastic carrier bag	Taped on, no Kapton windows
Paste	Toothpaste	Spatula in Kapton windows
Powder 1	Silver behenate	Spatula in Kapton windows
Powder 2	Fumed silicon	Spatula in (under fume hood) Kapton windows
Powder 3	Sodium chloride salt	Spatula in Kapton windows
Viscous liquid	Egg yolk	Decanted in Kapton windows
Viscous liquid 2	Egg white	Pipetted in (slow uptake) Kapton windows
