# Supplementary material for: Design of a multipurpose sample cell holder for the Diamond Light Source high-throughput SAXS beamline B21
Source: J Synchrotron Radiat. 2021 Jan 1;28(Pt 1):318–21. doi: 10.1107/S1600577520013831 (PMC7842227; doi:10.1107/S1600577520013831)
Supplement: Supplementary file 1 [file s-28-00318-sup1.pdf]

JOURNAL OF SYNCHROTRON RADIATION

Volume 28 (2021)

Supporting information for article:

Design of a multipurpose sample cell holder at a B21, Diamond Light Source BioSAXS beamline

Charlotte Jennifer Chante Edwards-Gayle, Nikul Khunti, Ian W. Hamley, Katsuaki Inoue, Nathan Cowieson and Robert Rambo

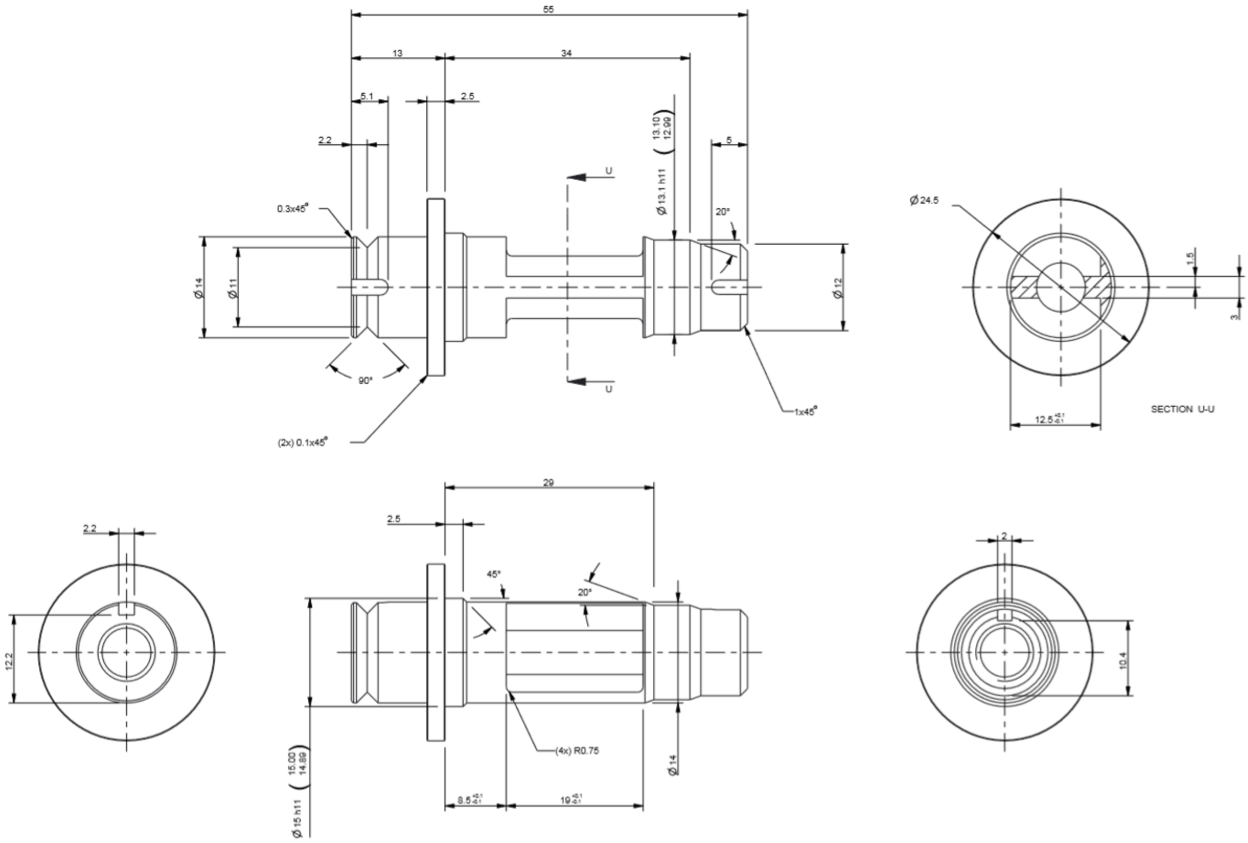

Figure S1. Technical Drawing of MPS Cell

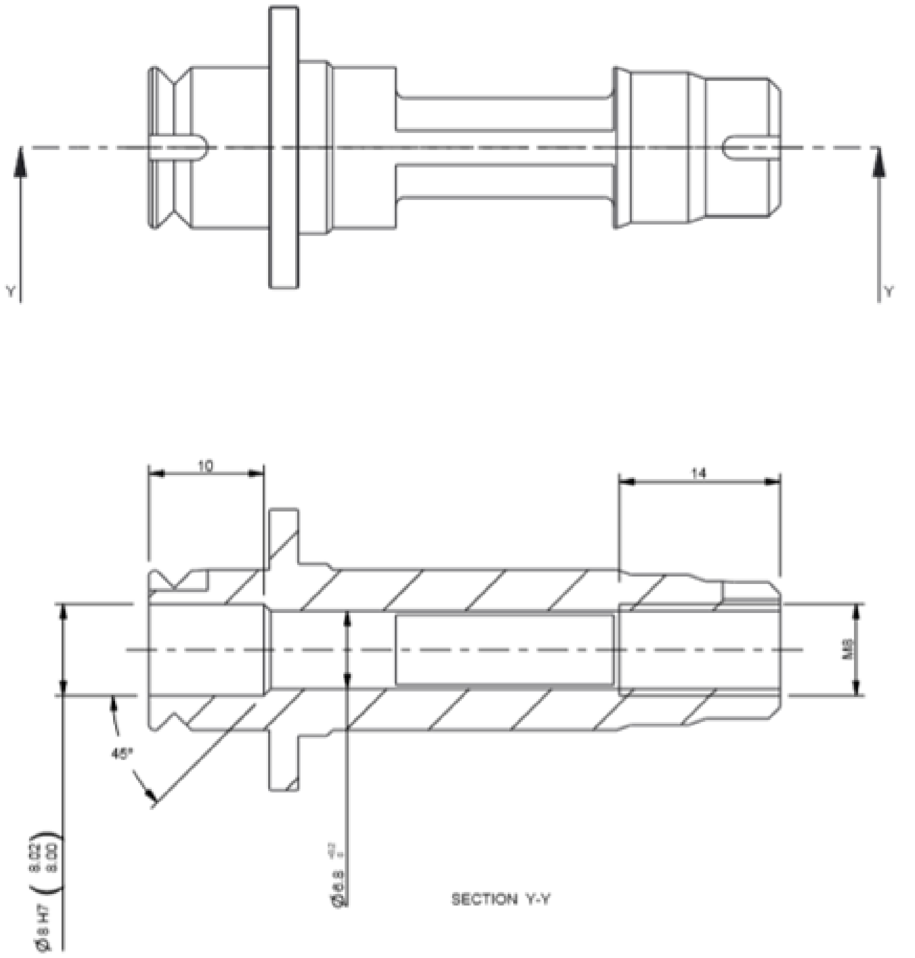

Figure S2. Technical Drawing of MPS Cell sample pod.

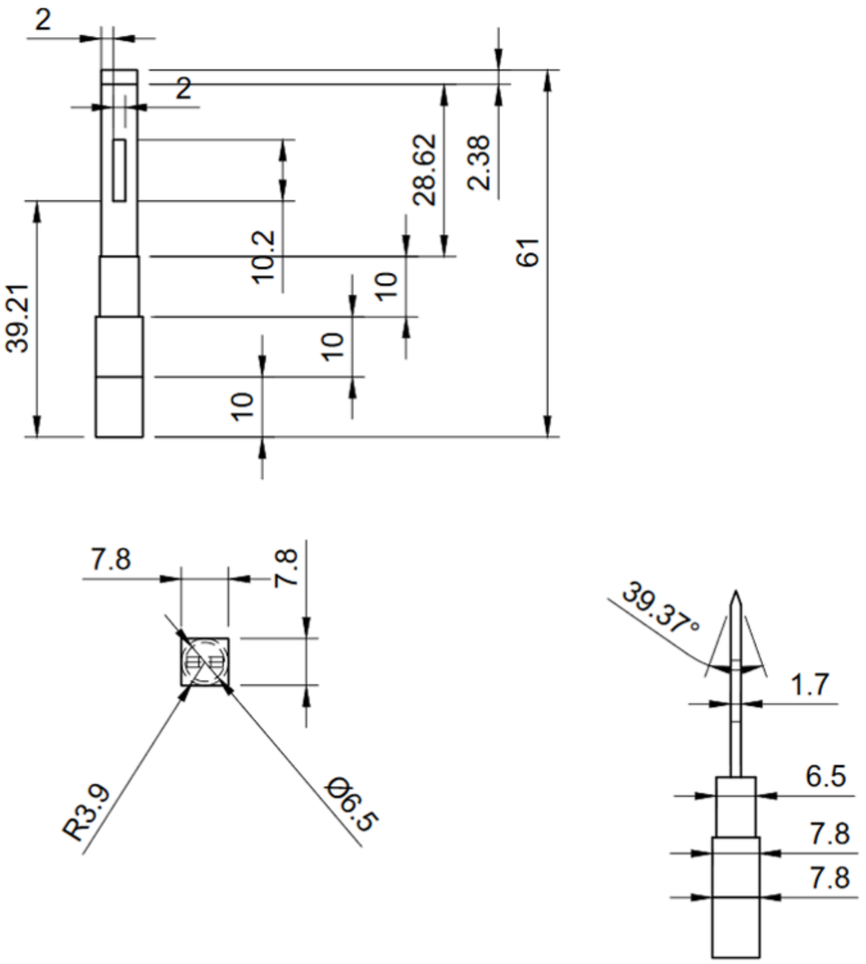

Figure S3. Technical drawing of the MPS Cell sample stick

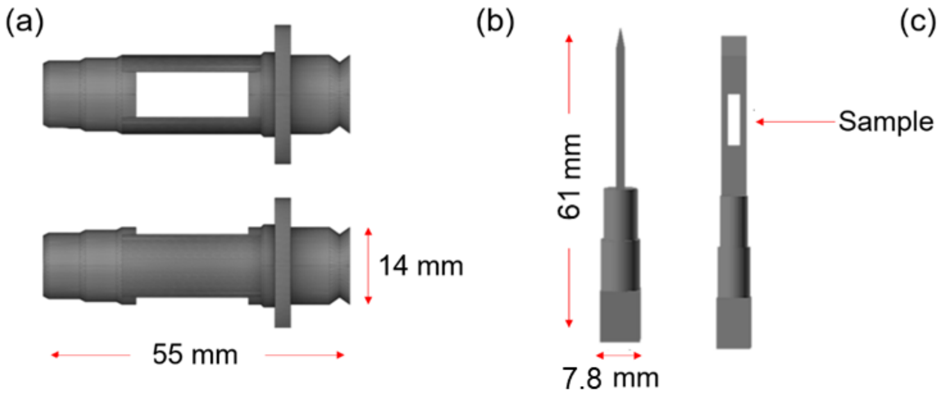

Figure S4. 3D CAD drawing of the MPS cell. a) shows two views of the Sample Pod. The Sample Pod is used in a vacuum environment and requires the attachment of windows along the X-ray beam path to separate the vacuum from the inner chamber that will hold the sample stick and b) Profile and side views of the Sample Stick. The rectangular opening supports non-liquid samples. c) Capillary insert.

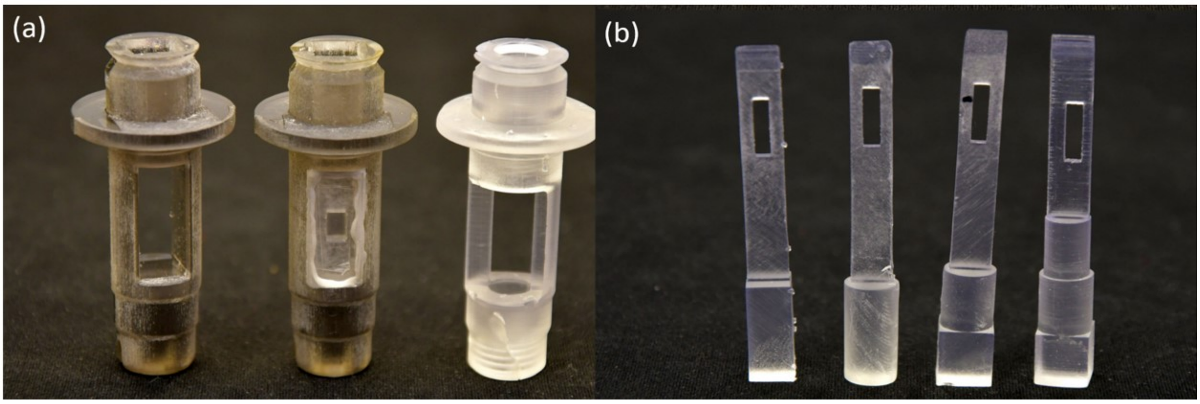

Figure S5. 3D printed prototypes of the MPS cell a) the sample pod, with the left being the first design, and the right hand side being the final design. The middle cell is the first cell with the frame glued in place using epoxy resin. and b) shows the evolution of design of the sample stick from the first designs (left) with a squared toped insert to the triangular coned insert with cylindrical neck, with the right hand side being the final design.

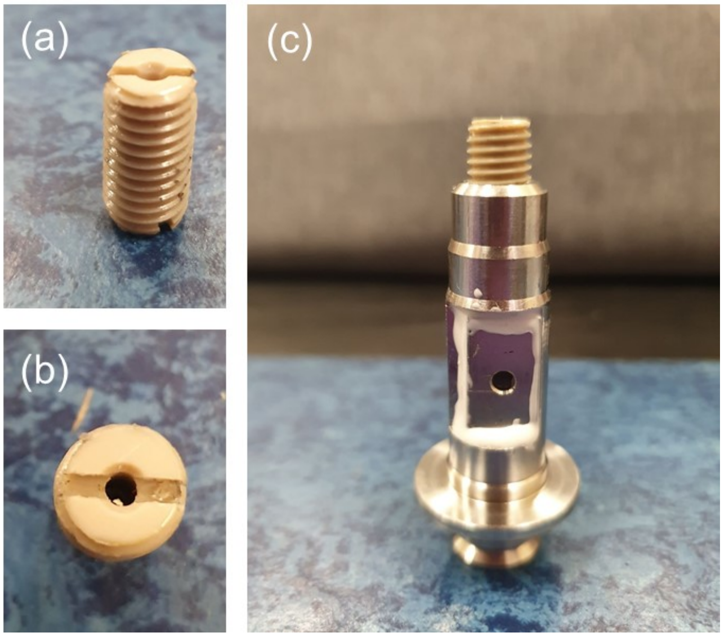

Figure S6. Different viewpoints of the alignment thread.
